# Supplementary material for: Transcriptional repression and enhancer decommissioning silence cell cycle genes in postmitotic tissues
Source: G3 (Bethesda). 2024 Aug 22;14(10):jkae203. doi: 10.1093/g3journal/jkae203 (PMC11457063; doi:10.1093/g3journal/jkae203)
Supplement: jkae203_Supplementary_Data [file jkae203_supplementary_data.zip › Supplemental_Figure_Legends_G3-2024-405265.docx]

**Supplement to: Transcriptional repression and enhancer decommissioning silence cell cycle genes in postmitotic tissues.**

Elizabeth A. Fogarty, Elli M. Buchert, Yiqin Ma, Ava Nicely, and Laura A. Buttitta^*^

Molecular, Cellular and Developmental Biology, University of Michigan, Ann Arbor 48109

^*^ Author for correspondence: buttitta@umich.edu

**Contains Legends for Figures S1-S14**

**Figure S1 STARR-Seq fails to identify many developmentally dynamic enhancer regions *in vivo.*** (**A-B**) STARR-Seq, ATAC-Seq, and ChIP-Seq data at the *CycE* (**A**) and *stg* (**B**) loci. Orange boxes indicate STARR-Seq housekeeping enhancers and magenta boxes indicate STARR-Seq developmental enhancers. ATAC-Seq accessibility data from wing, eye, and brain at L3, 24h APF, and 44h APF. ChIP-Seq data for Trl, DREF, M1BP, and Myc. Y-axes indicate the normalized read counts per million. HK, housekeeping. Dev, developmental. (**C**) Bar plots indicating the percent of ATAC-Seq peaks that overlapped housekeeping, developmental, or both types of STARR-Seq defined enhancers, grouped by those that showed statistically significant changes in ATAC-Seq intensity across the time course (Dynamic) versus those that did not (Static).

**Figure S2 Dynamic chromatin regions within *e2f1* function together to drive expression of *e2f1* transcript throughout the wing.** ATAC-seq shows chromatin accessibility in the wing during development. We observe that a few of these regions show enhancer activity in the wing. These enhancers may function in a modular fashion, as the sum of their expression patterns drive expression of *e2f1* throughout the pupal wing. The indicated Gal4 lines were crossed to G-TRACE with UAS-RFP (current expression) in magenta. DNA was stained with DAPI and shown in green. Note that intensity has been adjusted across each image to emphasize current expression. For intensity quantifications in Supp. Figs. 8 and 9 images were acquired under identical settings and intensity adjustments were not performed.

**Figure S3 Dynamic chromatin regions within *e2f1* show enhancer activity in the pupal eye.**

ATAC-seq data shows chromatin accessibility changes throughout development. The indicated Gal4 lines were crossed to G-TRACE with UAS-RFP (current expression) in magenta. DNA was stained with DAPI and shown in green. Note that intensity has been adjusted equally across entire images to emphasize current expression. For intensity quantifications in Figures S10 and S11, images were acquired under identical settings and intensity adjustments were not performed.

**Figure S4 Dynamic chromatin regions within *e2f1* show enhancer activity in the brain.**

ATAC-seq data shows chromatin accessibility changes throughout development. The indicated Gal4 lines were crossed to G-TRACE with UAS-RFP (current expression) in magenta. DNA was stained with DAPI and shown in green. Note that intensity has been adjusted equally across entire images to emphasize current expression. For intensity quantifications in Figures S10 and S11, images were acquired under identical settings and intensity adjustments were not performed.

**Figure S5 Modular enhancers function together to drive *stg* expression**

**throughout the larval and pupal wing.** ATAC-seq data shows dynamic chromatin accessibility changes throughout metamorphosis. The indicated Gal4 lines were crossed to G-TRACE with UAS-RFP (current expression) in magenta. DNA was stained with DAPI and shown in green. Note that intensity has been adjusted equally across entire images to emphasize current expression. For intensity quantifications in Figures S10 and S11, images were acquired under identical settings and intensity adjustments were not performed.

**Figure S6 Dynamic chromatin regions in the *stg* cis-regulatory region have**

**enhancer activity in the larval and pupal eye**.

Eye ATAC-seq data shows accessible chromatin throughout metamorphosis. The indicated Gal4 lines were crossed to G-TRACE with UAS-RFP (current expression) in magenta. DNA was stained with DAPI and shown in green. Note that intensity has been adjusted equally across entire images to emphasize current expression. For intensity quantifications in Figures S10 and S11, images were acquired under identical settings and intensity adjustments were not performed.

**Figure S7 Dynamic chromatin regions in the *stg* cis-regulatory region have**

**enhancer activity in the larval and pupal brain.**

The indicated Gal4 lines were crossed to G-TRACE with UAS-RFP (current expression) in magenta. DNA was stained with DAPI and shown in green. Note that intensity has been adjusted equally across entire images to emphasize current expression. For intensity quantifications in Figures S10 and S11, images were acquired under identical settings and intensity adjustments were not performed.

**Figure S8 Reporters driving dsGFP**

The indicated Gal4 lines were crossed to UAS-de-stabilized GFP (ds-GFP) in shown in greyscale. Tissue outlines are shown with yellow dotted lines. Quantifications of enhancer activity using dsGFP expression are shown. Images were acquired under identical settings without intensity adjustments. Quantifications were performed using Integrated intensity normalized to background in Image J.

**Figure S9 ‘Empty’ Janelia-Gal4 line shows some current expression in pupal eyes and brains.** The control Janelia Gal4 line without an inserted genomic fragment, containing the Drosophila synthetic Core promoter (DSCP) was crossed to G-TRACE with UAS-RFP (current expression) in red for overlays and greyscale in single channel images. Past lineage-tracing expression is in green in overlays and DNA was stained with DAPI and shown in blue in overlays. Note that intensity has been adjusted equally across entire images to emphasize current expression. We note significant past and current expression from the DSCP in pupal eyes and brains, which may confound enhancer quantifications at 24 and 44h APF.

**Figure S10**  **Quantifications of enhancer activity for the indicated *e2f1* Gal4 lines** The indicated Gal4 lines were crossed to G-TRACE with UAS-RFP (current expression) quantified. Images were acquired under identical settings without intensity adjustments. Quantifications were performed using Integrated intensity normalized to background in Image J.

**Figure S11 Quantifications of enhancer activity for the indicated *stg* Gal4 lines.** The indicated Gal4 lines were crossed to G-TRACE with UAS-RFP (current expression) quantified. Images were acquired under identical settings without intensity adjustments. Quantifications were performed using Integrated intensity normalized to background in Image J.

**Figure S12 Most cell cycle gene enhancers remain accessible across many postmitotic mammalian cell types.** A variety of mammalian differentiation models support the postmitotic maintenance of chromatin accessibility at cell cycle genes in vivo and in vitro. (A) Line plot showing average DNase-Seq signal at peaks (+/- 1 kilobase from peak center) associated with all cell cycle genes in purified cardiomyocytes from adult (9 weeks old) mouse. Data generated by (El-Nachef et al. 2018) and accessed from the European Nucleotide Archive, Bioproject accession number PRJNA449942. (B) Line plot showing average DNase-Seq signal at peaks (+/- 1 kilobase from peak center) associated with all cell cycle genes in whole mouse heart at embryonic day 10.5 (E10.5, pink), postnatal day 0 (P0, red), and 8 week old adult (dark red). Data generated by the ENCODE project; accession numbers ENCFF001OIQ, ENCFF001PMV, ENCFF069XLI, ENCFF215XVP, ENCFF268IOJ, ENCFF400CRF, ENCFF464YJZ, ENCFF610LLQ, ENCFF807ZAM, ENCFF938AEP. (C) Line plot showing average ATAC-Seq signal at peaks (+/- 1 kilobase from peak center) associated with repressed cell cycle genes in human keratinocytes at day 0 (light green), day 3 (green), and day 6 (dark green) of calcium-induced differentiation in vitro. RNA-Seq data from the same time points were used to define repressed cell cycle genes (genes with decreasing expression values across each sequential time point). Data generated by the ENCODE project and accessed via NCBI’s Sequence Read Archive; ATAC-Seq accession numbers SRR14305300 thru SRR14305309, SRR14305683 thru SRR14305692, SRR14305769 thru SRR14305776; RNA-Seq accession numbers SRR14832907, SRR14832908, SRR14833794, SRR14833795, SRR14828962, SRR14828963. (D) Line plot showing average ATAC-Seq signal at peaks (+/- 1 kilobase from peak center) associated with repressed cell cycle genes in mouse C2C12 cells in growth conditions (light purple), and after 24 hours (purple) and 72 hours (dark purple) of serum starvation-induced differentiation in vitro. RNA-Seq data from the same time points were used to define repressed cell cycle genes (genes with decreasing expression values across each sequential time point). Data generated by (Harada et al. 2018) and accessed from NCBI GEO Accession GSE104389.

**Figure S13 Loci encoding the orthologs of Stg and CycE provide no obvious indication of postmitotic enhancer decommissioning in the developing mouse retina.** ATAC-Seq data tracks from the mouse retina at the *Cdc25c* locus (**A**, ortholog of Stg in flies) and the Ccne2 locus (**B**, ortholog of CycE in flies).

**Figure S14 Loci encoding the Myc family members provide evidence of postmitotic enhancer decommissioning in the developing mouse retina.** (**A-C**) ATAC-Seq data tracks from the mouse retina at the *Myc* gene body (**A**), at the topologically associating domain (TAD) containing *Myc* (**B**), and the *Mycl* locus (**C**). (**D**) Heatmap depicting the average transcript expression values for Myc family genes. Data are presented as normalized Log2 Count Per Million (CPM) values.

**References**

El-Nachef, D., K. Oyama, Y.Y. Wu, M. Freeman, Y. Zhang *et al.*, 2018 Repressive histone methylation regulates cardiac myocyte cell cycle exit. *J Mol Cell Cardiol* 121:1-12.

Harada, A., K. Maehara, Y. Ono, H. Taguchi, K. Yoshioka *et al.*, 2018 Histone H3.3 sub-variant H3mm7 is required for normal skeletal muscle regeneration. *Nat Commun* 9 (1):1400.
