# Supplementary figures and images for: Transcriptional repression and enhancer decommissioning silence cell cycle genes in postmitotic tissues
Source: G3 (Bethesda). 2024 Aug 22;14(10):jkae203. doi: 10.1093/g3journal/jkae203 (PMC11457063; doi:10.1093/g3journal/jkae203)

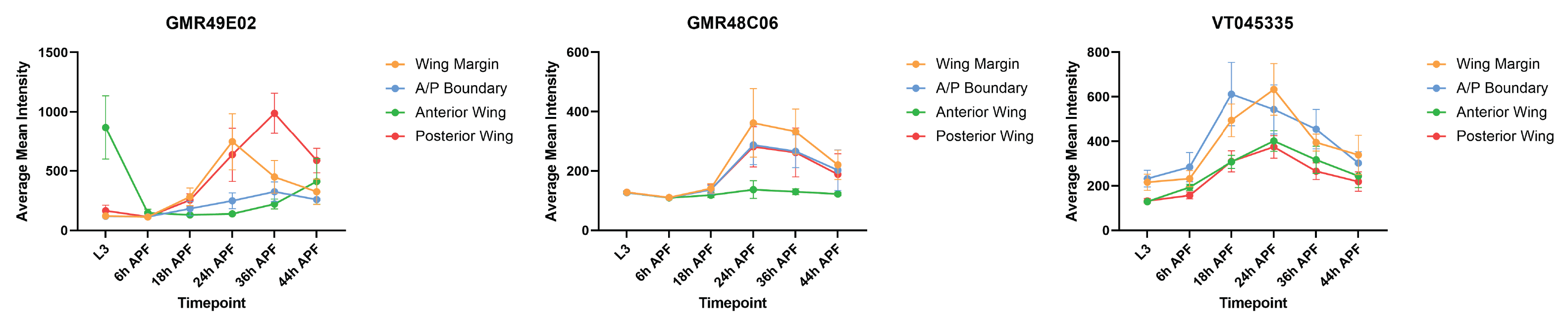

Supplement: jkae203_Supplementary_Data [file jkae203_supplementary_data.zip › Supplemental_Figure_S10_G3-2024-405265.png]

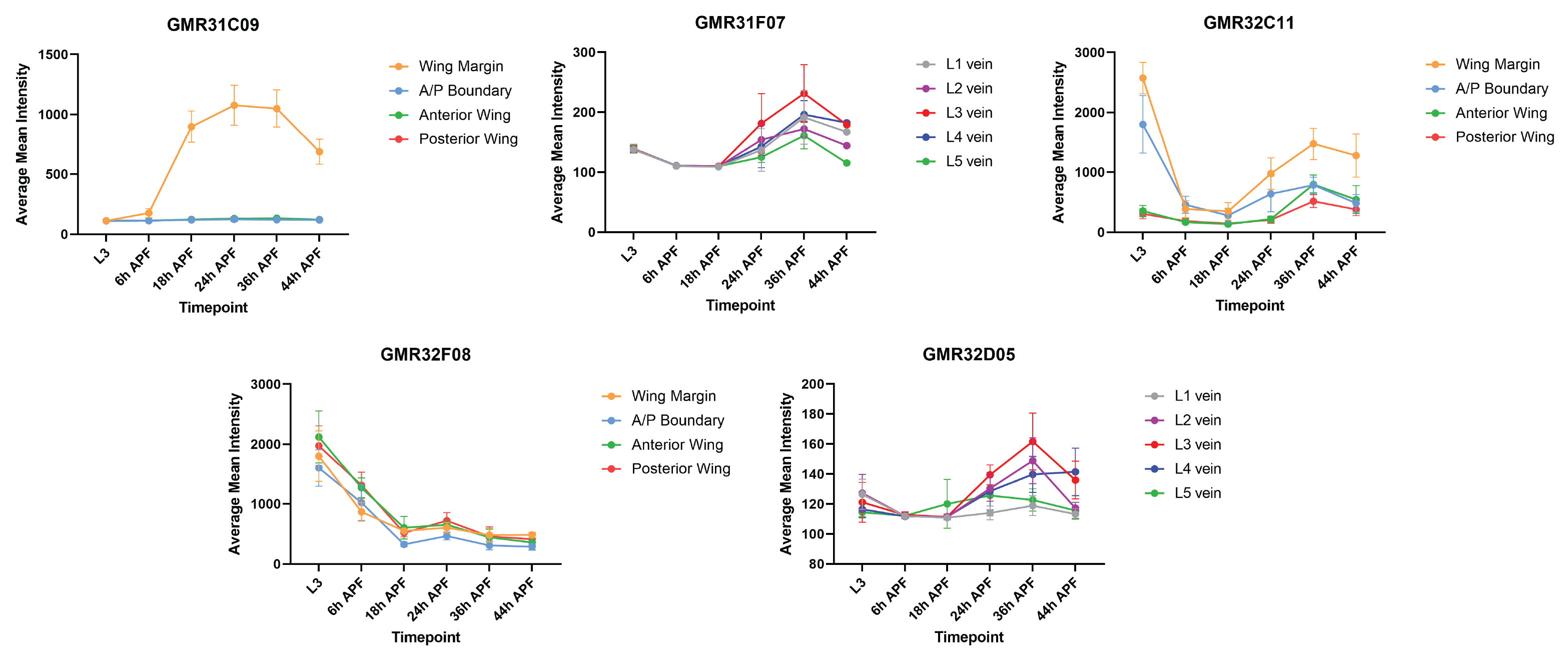

Supplement: jkae203_Supplementary_Data [file jkae203_supplementary_data.zip › Supplemental_Figure_S11_G3-2024-405265.png]

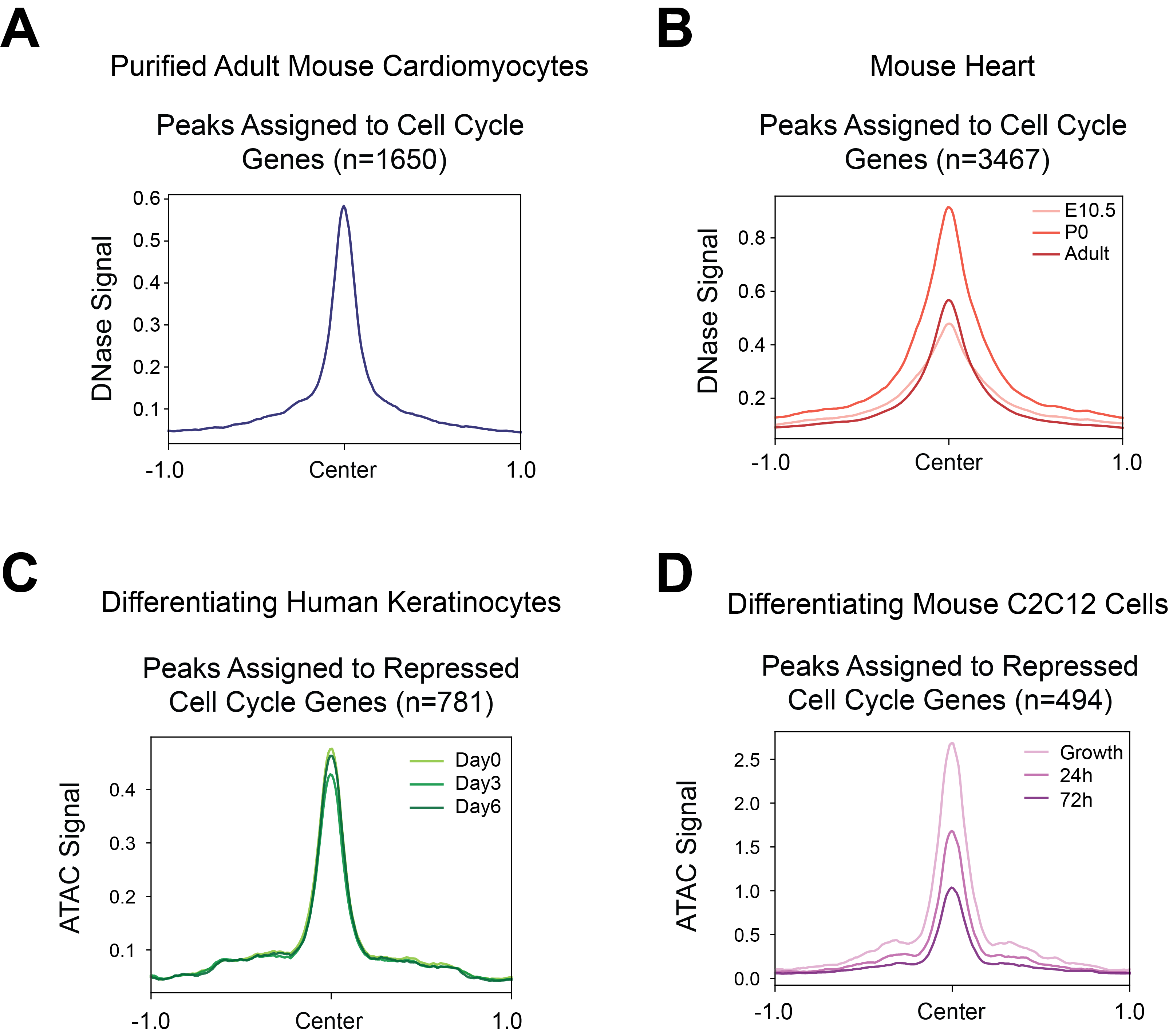

Supplement: jkae203_Supplementary_Data [file jkae203_supplementary_data.zip › Supplemental_Figure_S12_G3-2024-405265.png]

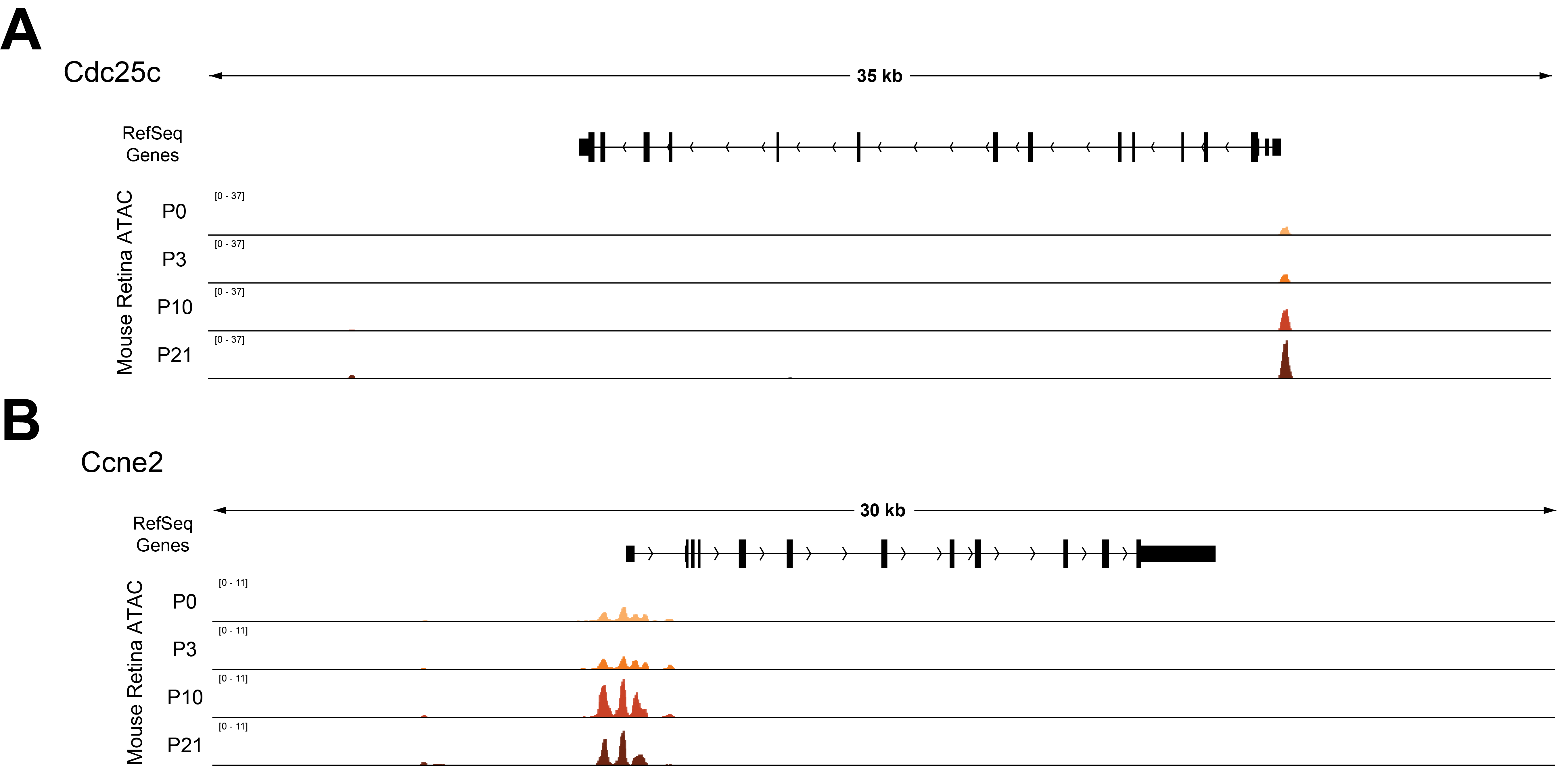

Supplement: jkae203_Supplementary_Data [file jkae203_supplementary_data.zip › Supplemental_Figure_S13_G3-2024-405265.png]

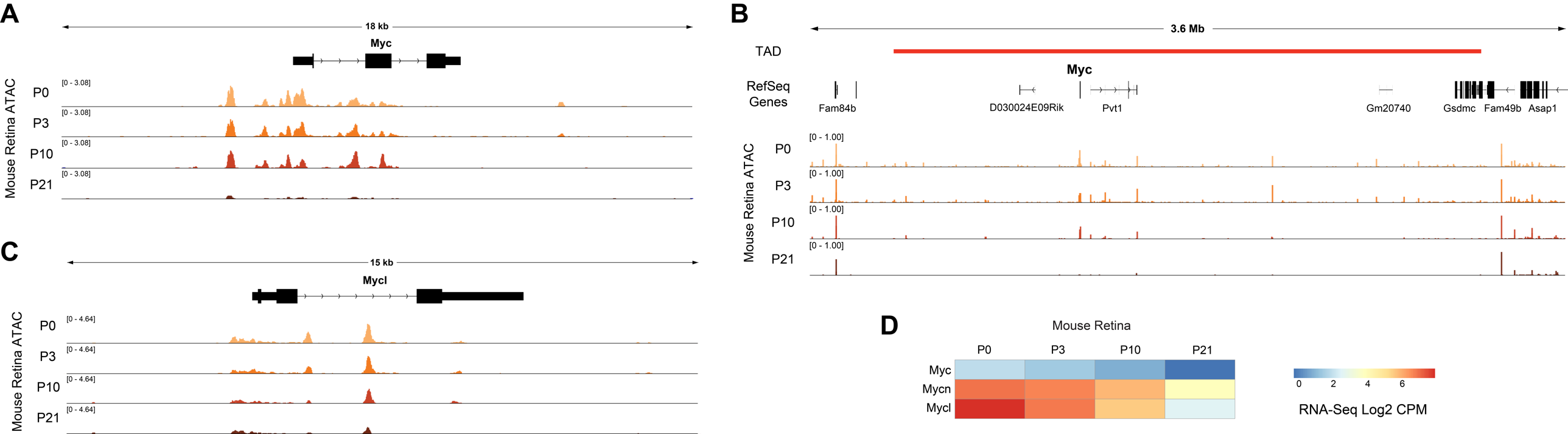

Supplement: jkae203_Supplementary_Data [file jkae203_supplementary_data.zip › Supplemental_Figure_S14_G3-2024-405265.png]

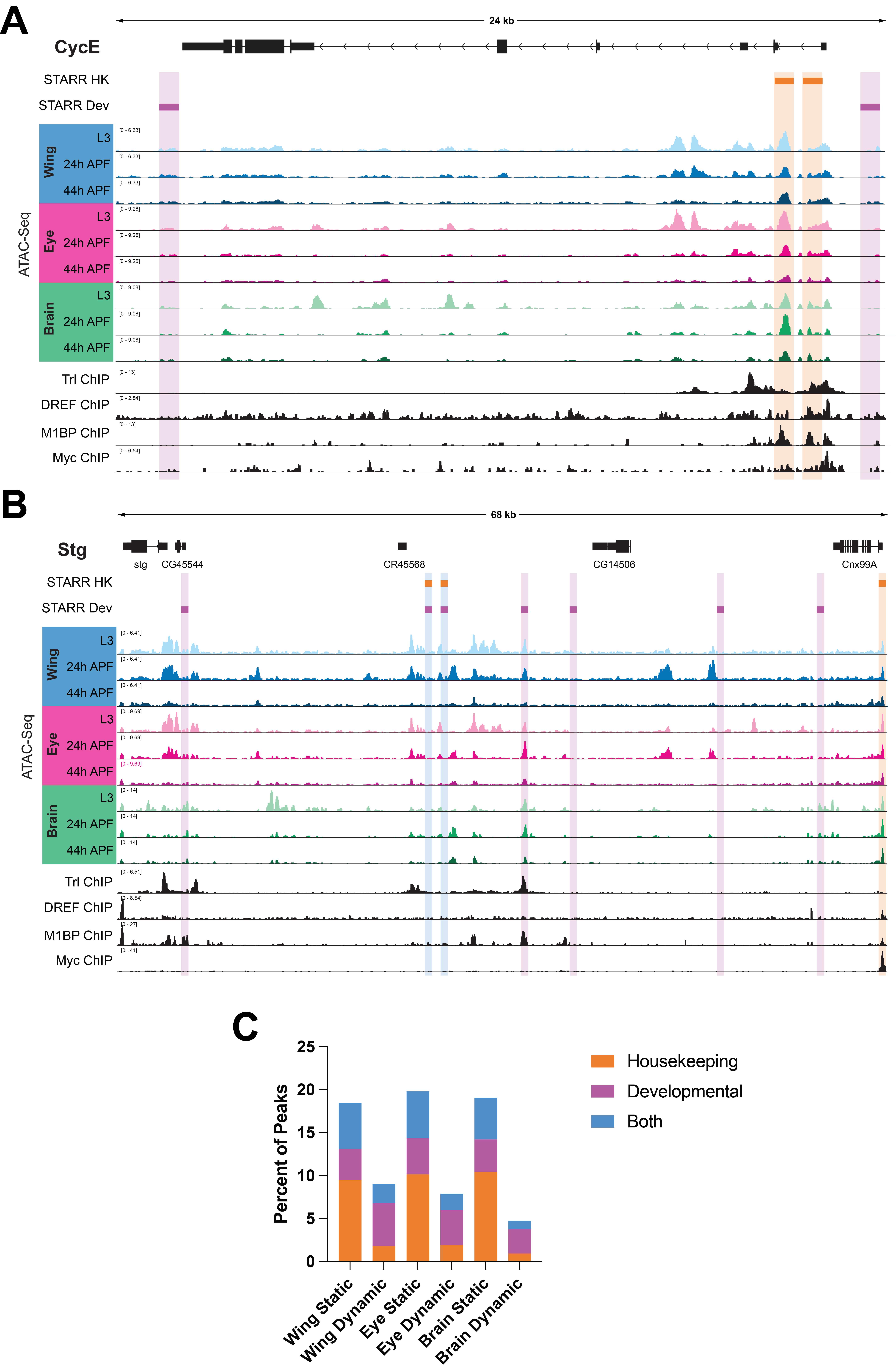

Supplement: jkae203_Supplementary_Data [file jkae203_supplementary_data.zip › Supplemental_Figure_S1_G3-2024-405265.png]

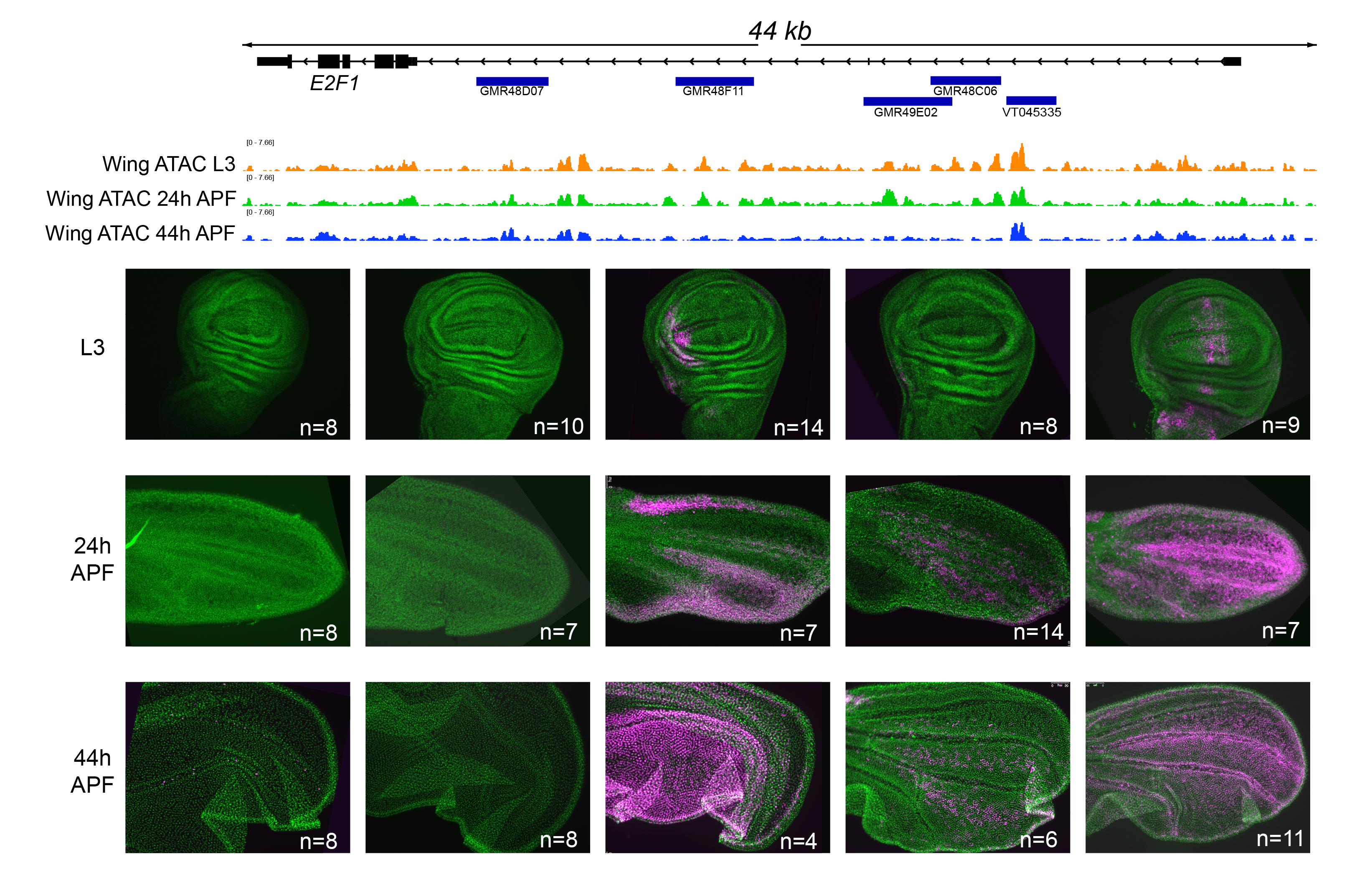

Supplement: jkae203_Supplementary_Data [file jkae203_supplementary_data.zip › Supplemental_Figure_S2_G3-2024-405265.png]

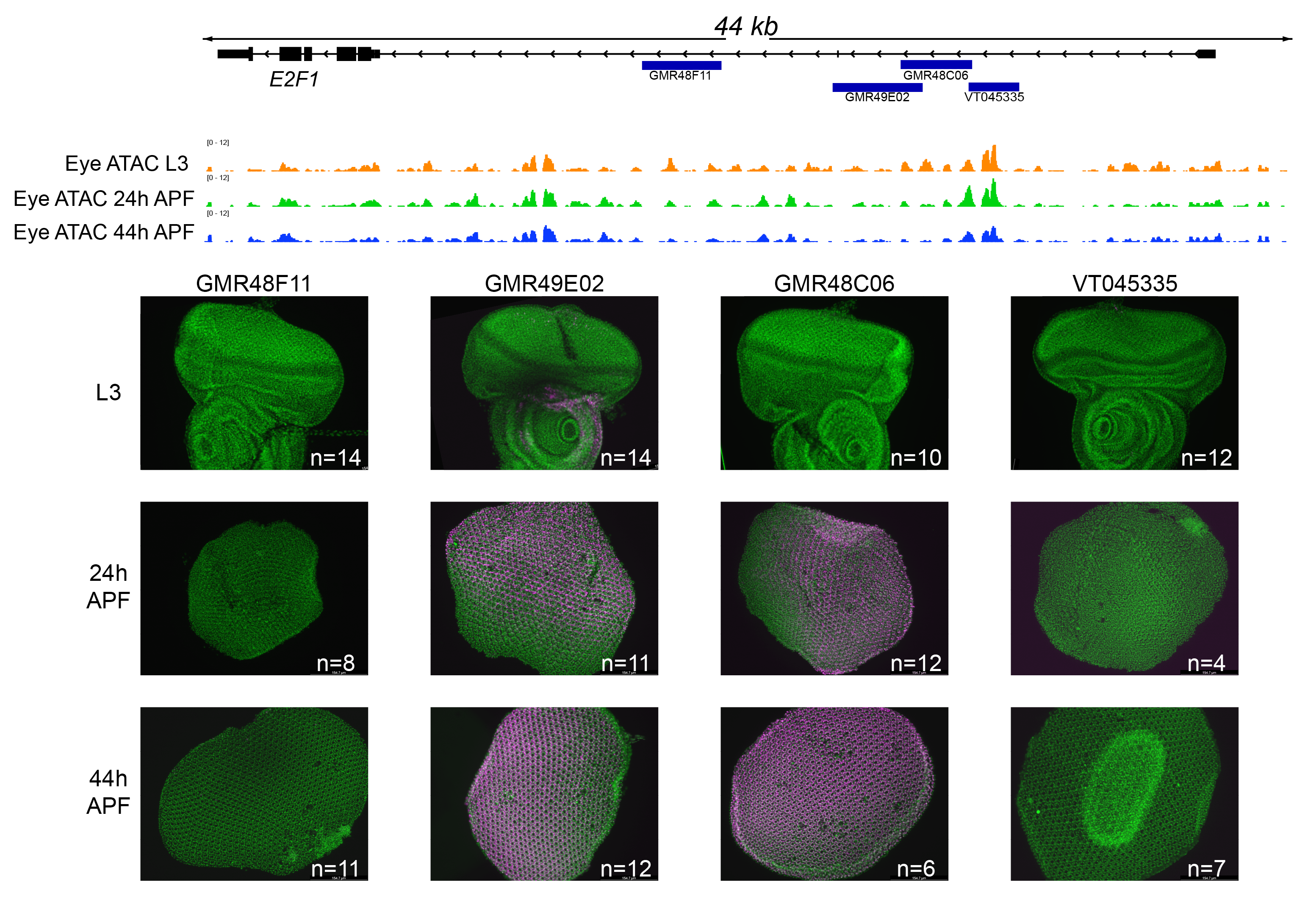

Supplement: jkae203_Supplementary_Data [file jkae203_supplementary_data.zip › Supplemental_Figure_S3_G3-2024-405265.png]

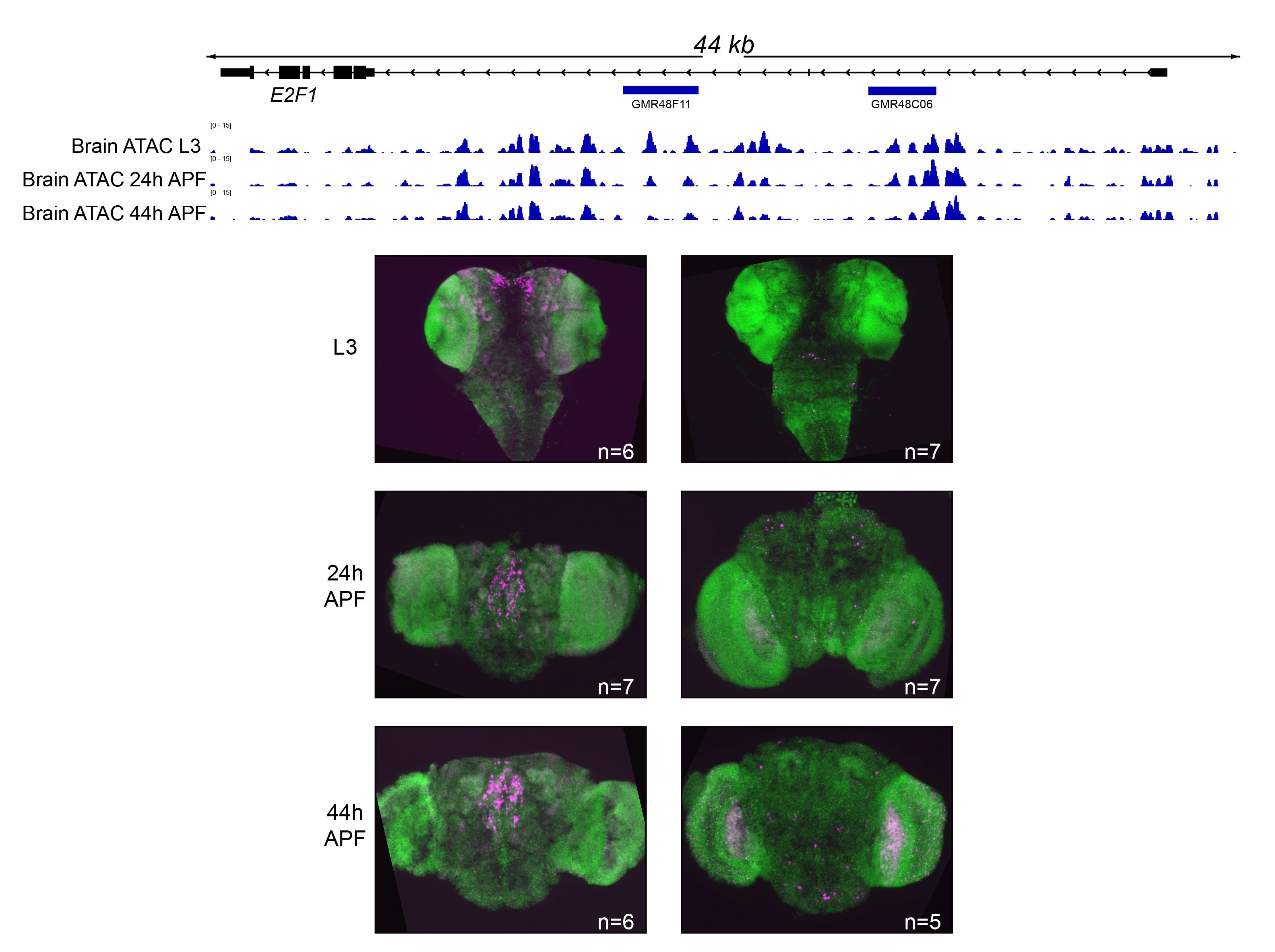

Supplement: jkae203_Supplementary_Data [file jkae203_supplementary_data.zip › Supplemental_Figure_S4_G3-2024-405265.png]

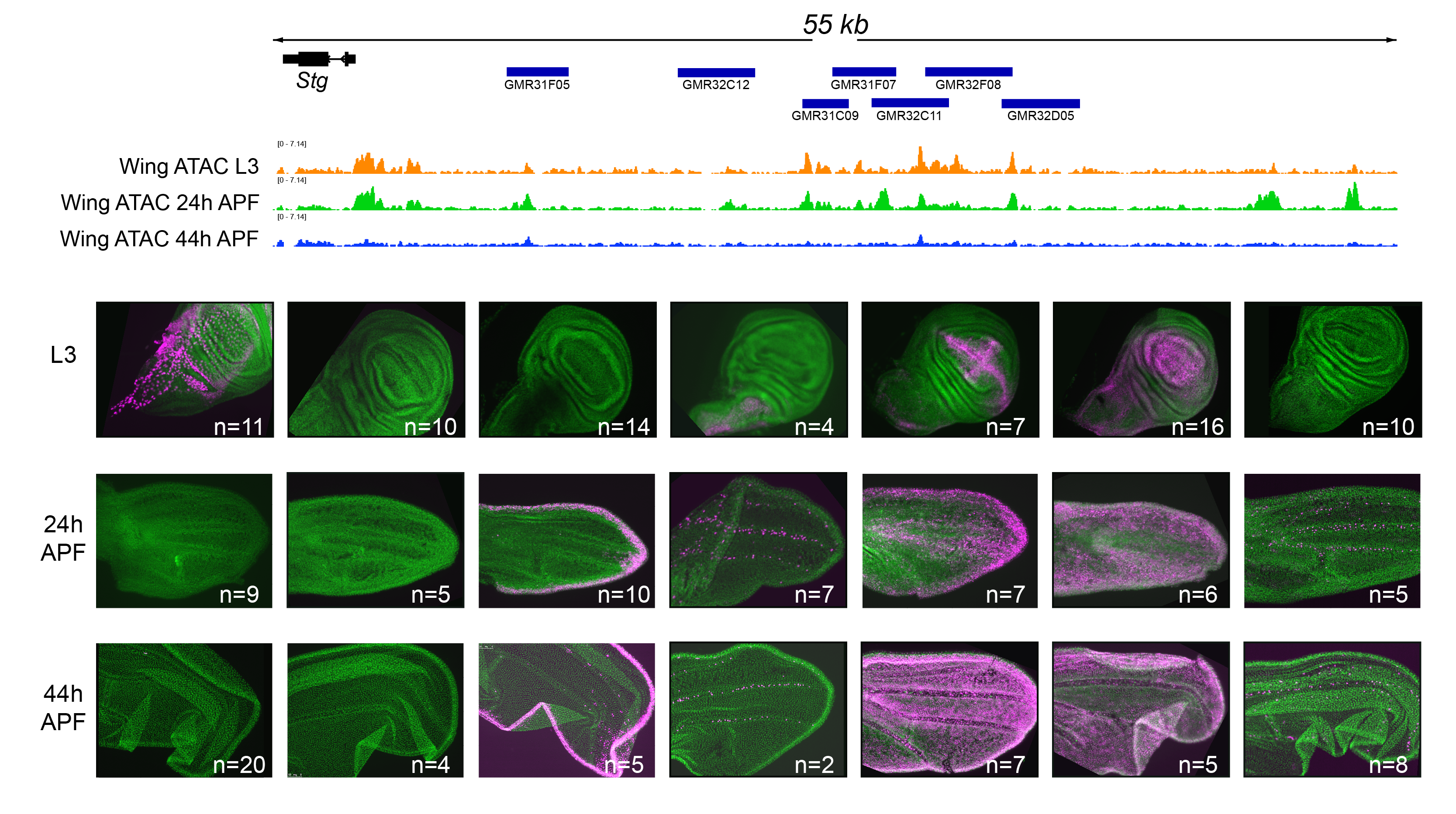

Supplement: jkae203_Supplementary_Data [file jkae203_supplementary_data.zip › Supplemental_Figure_S5_G3-2024-405265.png]

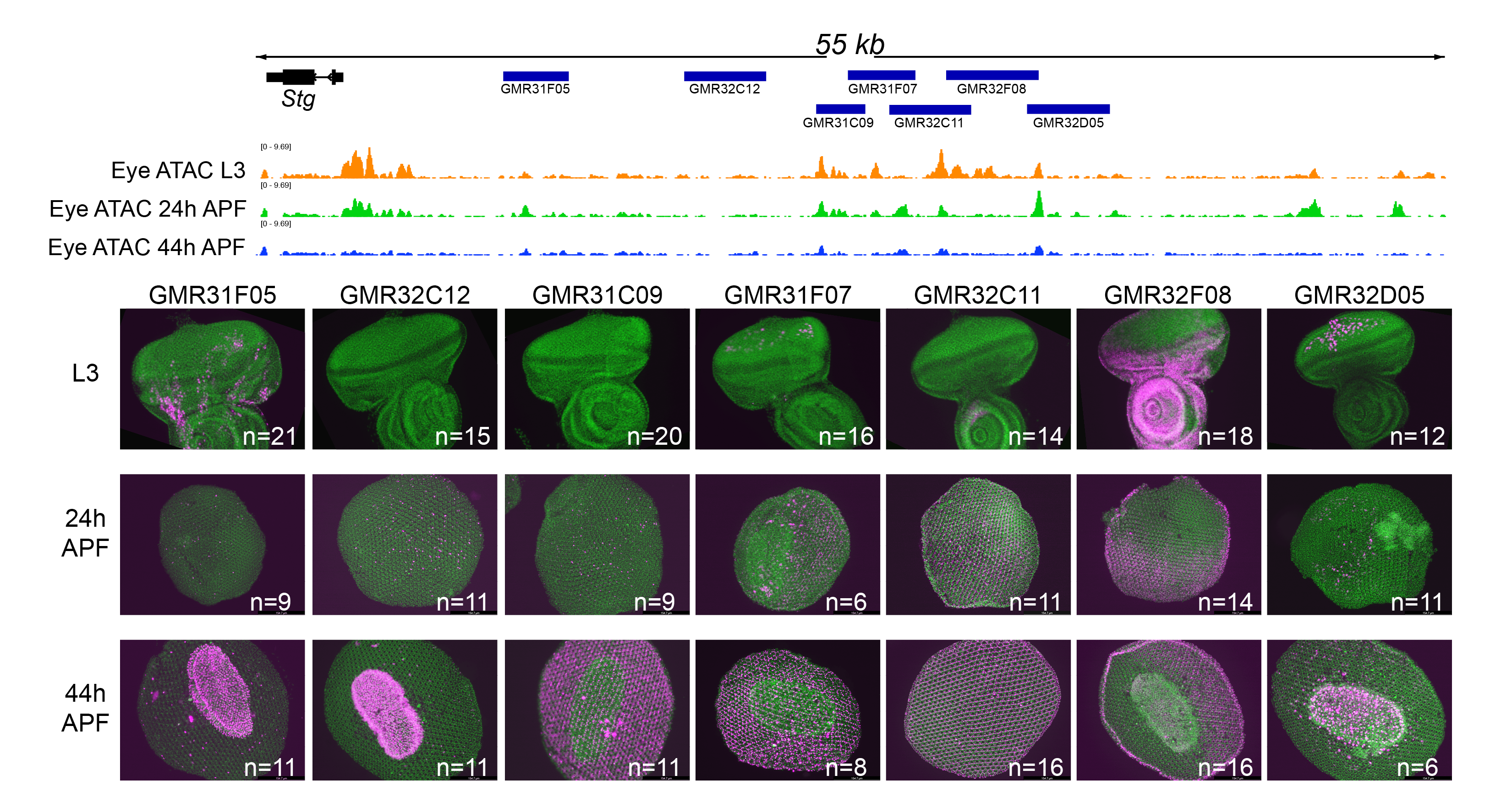

Supplement: jkae203_Supplementary_Data [file jkae203_supplementary_data.zip › Supplemental_Figure_S6_G3-2024-405265.png]

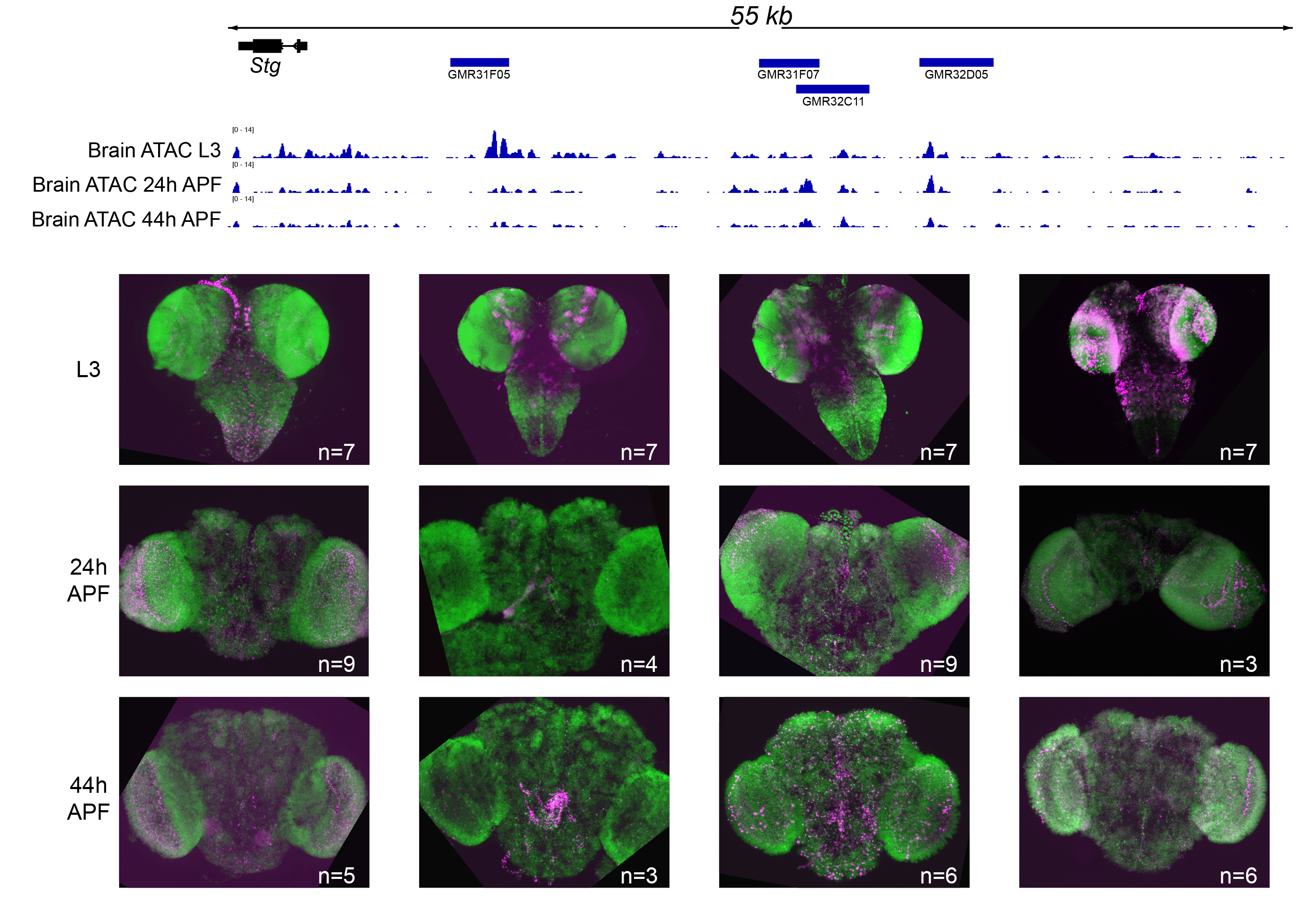

Supplement: jkae203_Supplementary_Data [file jkae203_supplementary_data.zip › Supplemental_Figure_S7_G3-2024-405265.png]

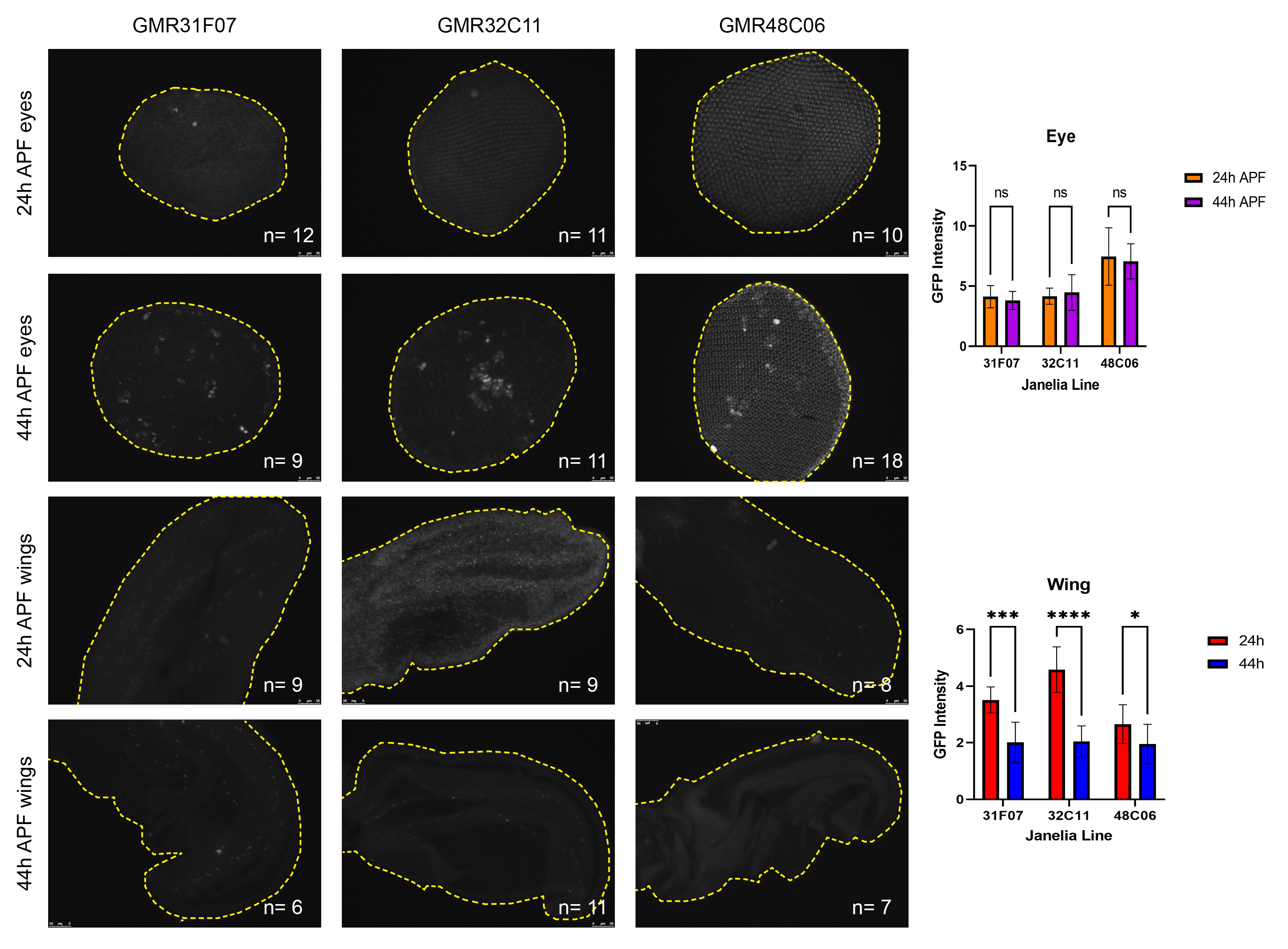

Supplement: jkae203_Supplementary_Data [file jkae203_supplementary_data.zip › Supplemental_Figure_S8_G3-2024-405265.png]

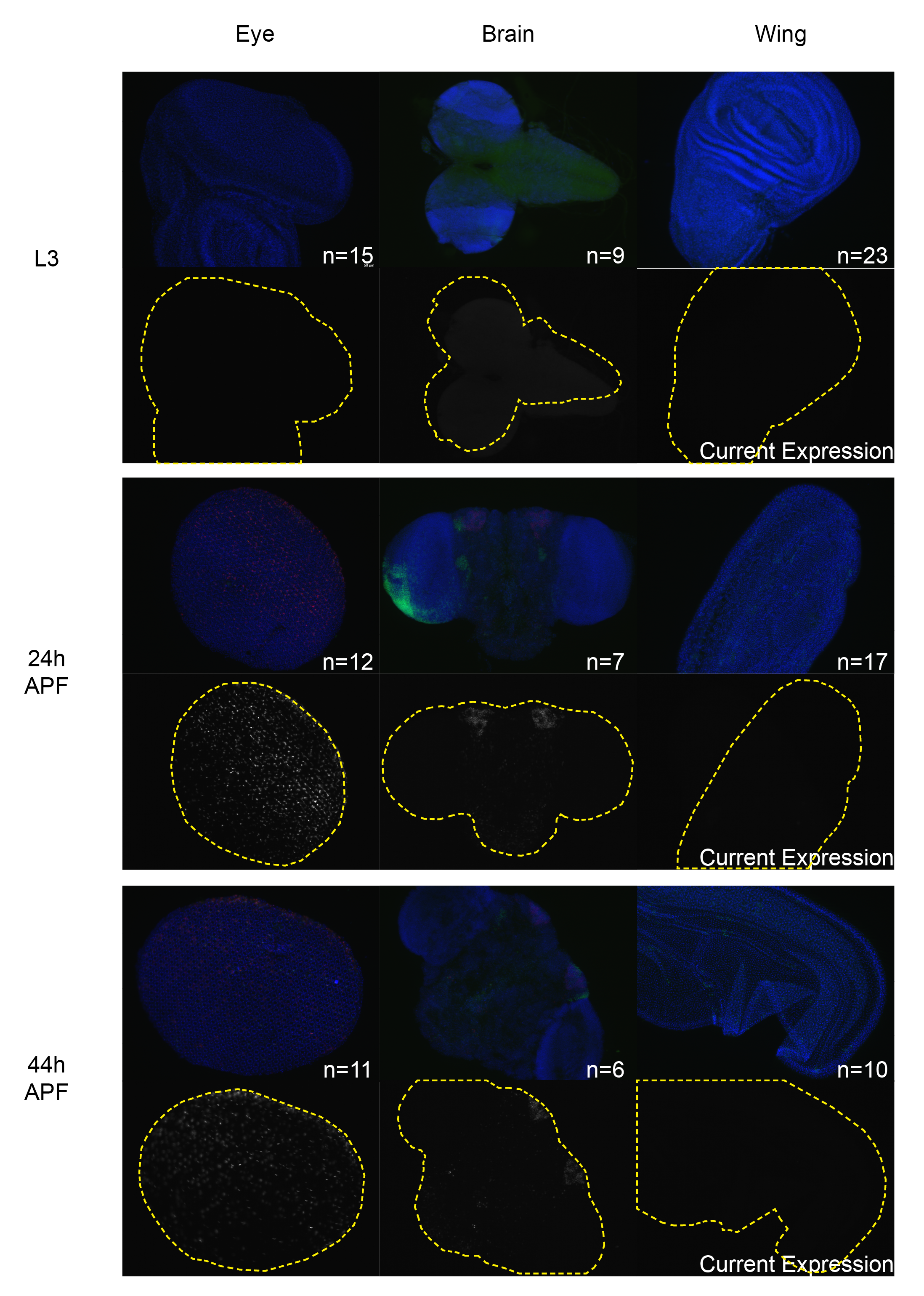

Supplement: jkae203_Supplementary_Data [file jkae203_supplementary_data.zip › Supplemental_Figure_S9_G3-2024-405265.png]
